# Supplementary material for: Mapping the gene regulatory landscape of archaic hominin introgression in modern Papuans
Source: PLoS Genet. 2026 Mar 24;22(3):e1012067. doi: 10.1371/journal.pgen.1012067 (PMC13012733; doi:10.1371/journal.pgen.1012067)
Supplement: Attachment — Submitted filename: Response to reviewers.pdf [file pgen.1012067.s037.pdf]

Reviewer #1: The authors have addressed all my remarks adequately and have significantly strengthened their statistical analyses and the manuscript as a whole.

My only remaining concern relates to the updated figure 3, and the associated statements. « We were able to identify haplotypes for which all alleles and corresponding single variant sequences were active, but were the combination of allele showed greater activity than the single variant sequence alone (Figure 3B), suggesting addivity »

If I understand figure 3B correctly, the authors have actually tested 8 sequences for the pair of variants located at chr6 :107,746,260 & chr6 :107,746,429 ie. 169 bp away from each other.
- Two 170 bp sequences centered on 107,746,260 : (i.e. from ~107,746,175 to 107,746,345 bp), called SNP1-centered sequences 1H & 1D on the figure 3B
- Two 170 bp sequences centered on 107,746,429 : (i.e. from ~107,746,344 to 107,746,514 bp), called SNP2-centered sequences 2H & 2D on the figure 3B
- Four 170bp sequences containing each combination of alleles from both variants: (i.e. from ~107,746,260 to 107,746,430 bp) callled haplotypic sequences HH, HD, DH, and DD on the figure 3B.

The reviewer is correct that we have tested 8 sequences for the pair of variants in Figure3B, but we apologise that the coordinates are misleading. The coordinates shown on Figure3B-F reflect the coordinates of start and end of haplotype-like sequence, not the position of the individual SNPs included within the haplotype-like sequence. For clarity, we have updated Figure3B-F to now show the coordinates of the SNPs contained within the haplotype-like sequence, as well as the distance between the SNPs. The updated version of Figure 3 is reproduced below:

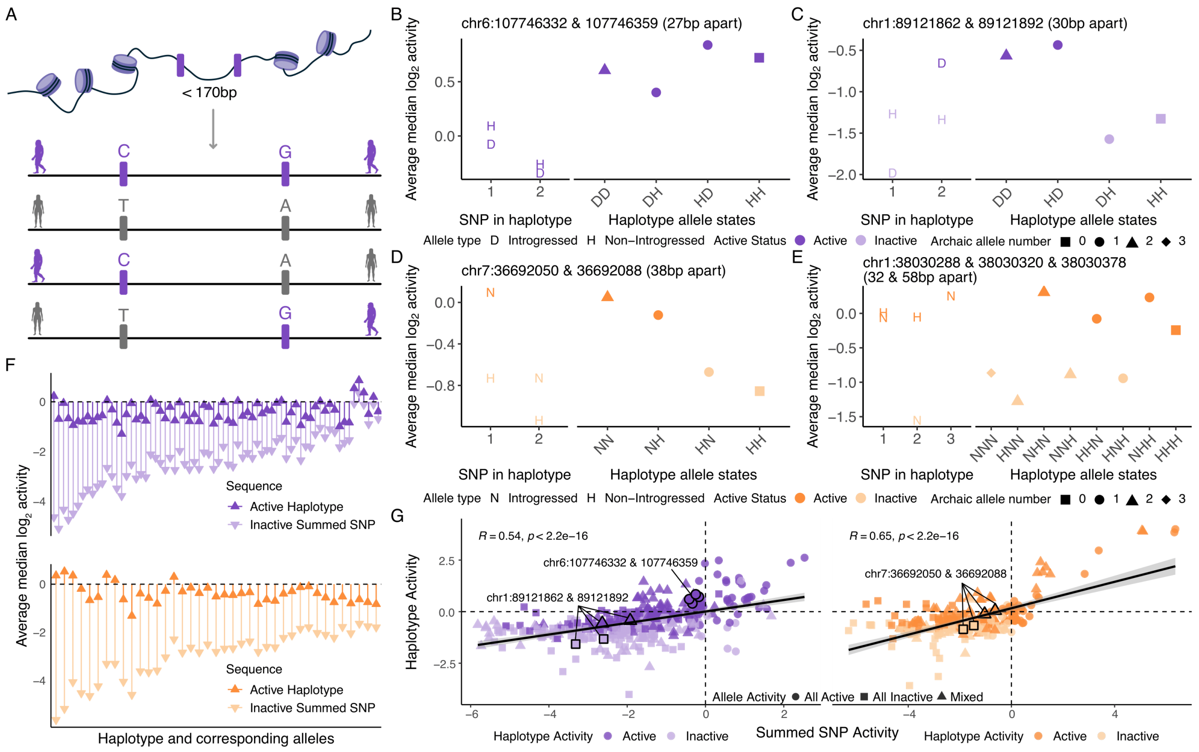

The caption for Figures 3B-E now reads:

"B-E.) Interactive effects between single variant sequences in haplotype-like sequences. Coordinates (in hg38) show the position of the SNPs included in haplotype-like sequences."

If that's correct, the authors can/should actually answers two separate questions :
1- Is the effect of each variant dependent on its position on the tested sequence ?
a. is (1D-1H), the effect of SNP1 positionned at the center of the tested sequence the same as (DH-HH) the effect of SNP1 positionned at the border of the tested (human) sequence?
b. is (2D-2H), the effect of SNP2 positionned at the center of the tested sequence the same as (HD-HH) the effect of SNP2 positionned at the border of the tested (human) sequence?

To test whether the effect of each variant is dependent on its position in the tested sequence, we asked how often single-variant sequences were designated with the same active state (active or inactive) with their corresponding haplotype-like sequences (e.g. single-variant sequence 1D and haplotype-like sequence DH). We find that single-variant and haplotype-like sequences are generally concordant in their activity (82-88% concordance), and that the frequency of concordance differed across allelic states (archaic-human, human-archaic and human-human). We have added these results to the text, lines 286-303 now read:

"We asked how often single-variant sequences showed concordant activity (active or inactive) with their corresponding haplotype-like sequences. We considered only haplotype-like sequences containing two SNPs for which all corresponding single-variant sequences were present in the final library. Additionally, we excluded haplotype-like sequences containing two archaic alleles as no corresponding single-variant sequence exists (single-variant sequences were constructed on a modern human background). This resulted in 2,115 single-variant and haplotype-like sequence pairs for testing. Across allelic states (archaic-human, human-archaic and human-human), most single-variant and haplotype-like sequence pairs shared the same active state (82-88% concordance), indicating that activity is generally robust to positional shifts of an allele within the tested sequence. However, the frequency of concordance differed significantly across allelic states ($\chi 2 = 14.442$, $p = 0.0007$). Sequences containing both archaic and modern alleles (archaic-human and human-archaic sequences) showed similar concordance rates (88.3% and 88.22% respectively), whereas sequences containing two modern alleles showed a decrease in concordance (82.26%). These results suggest that while single-variant and haplotype-like sequences are broadly consistent in their activity, modern human alleles may be more sensitive to positional or sequence context than archaic alleles."

2- Do archaic variants exhibit non-additive effect on gene expression ?
a. is (DH-HH) the effect of SNP1 on a human background, the same as (DD-HD), the effect of the effect of SNP1 on an archaic background ?

b. is (HD-HH) the effect of SNP2 on a human background, the same as (DD-DH), the effect of the effect of SNP2 on an archaic background ?

To test whether the activity of a given SNP was dependent on its background sequence, we asked how often haplotype-like sequences containing two archaic alleles (e.g. haplotype-like sequence DD) were designated with the same active state (active or inactive) with sequences containing one archaic allele (e.g haplotype-like sequences DH and HD). We find that concordance in activity is high for both comparisons (e.g. DD-DH and DD-HD), and that concordance rate did not significantly differ between the two comparisons. We have added these results to the text, lines 304-312 now read:

"For haplotype-like sequences containing two SNPs, we asked how often sequences containing two archaic alleles (archaic-archaic sequences) showed concordance in activity with sequences containing one archaic allele (archaic-human and human-archaic sequences) to test the effect of background sequence context. Concordance in activity was high in both comparisons (archaic-archaic:archaic-human = 95%, archaic-archaic:human-archaic = 93.9%). Concordance rates did not differ significantly between the two comparisons ($\chi 2 = 0.90$, $p = 0.34$), suggesting that activity is largely robust to the haplotype sequence context, and the presence of an additional archaic allele does not substantially alter regulatory activity."

What Is see from figure 3B would suggest that in the haplotypic sequences, SNP1 and SNP2 act in an additive manner, with SNP1 decreasing expression and SNP2 increasing expression, but the effect of SNP2 is position-dependent and is not detected in the SNP2-centered comparison (proper statistical testing for differntial activity would however be in order here). I think however that stating that « the combination of allele showed greater activity than the single variant sequence » is misleading, as a suggests a GxG interaction which is not supported by the data in this case.

We have rephrased our description of Figure 3B to avoid misrepresentation of the data. Lines 262-263 now read:

"We were able to identify haplotypes for which all alleles and corresponding single-variant sequences were active, but showed variation in activity levels (Figure 3B)"

Overall, given the comment to reviewer 2 regarding the difficulty to interpret off diagonal points in Figure 3G, I wonder if it would not be more staightforward to focus on haplotypic constructs, and compare for each variant, the effect on the human background vs effect on the archaic background (Question 1). A comparison of SNP effects between SNP-centrered or (human) haplotypic contructs (Question 2) could also be shown as a word of caution on how the design of the sequence construct may affect the inferred effect of tested variants.

Finally, it would be nice to highlight in figures 3F, 3G and S17 the specific SNPS that the authors choose to highlight in the Figure 3B-3E.

We have highlighted the haplotypes shown in Figure 3B-3D on Figure 3G. Note that the haplotype shown in 3F is not highlighted in Figure 3G, as only haplotypes for which the full set of corresponding single SNP oligos are shown in Figure 3G. Similarly, in Figure 3F only haplotypes for which all corresponding single SNP oligos are inactive, so the examples in Figure 3B-3F are not shown.

The updated version of Figure 3 is reproduced below:

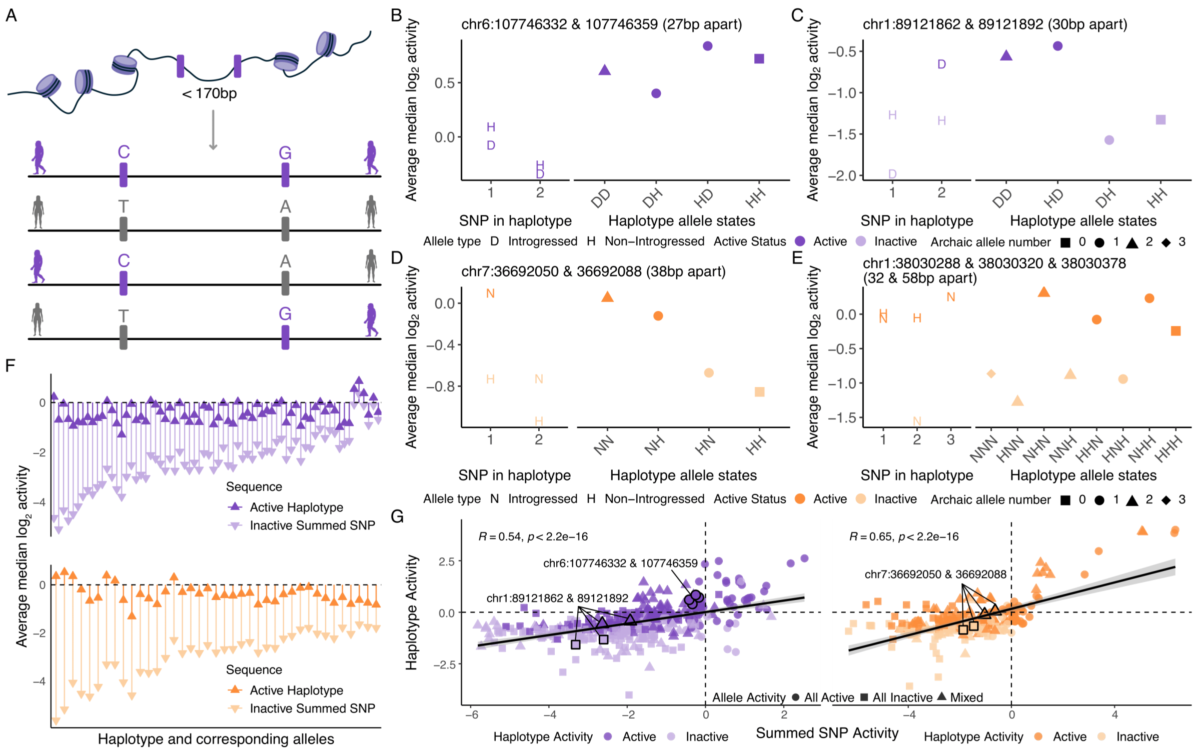

The caption for Figure 3G now reads:

"G.) Correlation between activity of haplotype-like sequences and the summed activity of the corresponding single SNP sequences for Denisovan (left) and Neanderthal (right) sequences, for haplotype-like sequences for which all corresponding single SNP sequences were present in the final library. Mixed activity of summed single SNP sequences represents cases where the single SNP sequences for a given haplotype sequence were different in their activity status. The haplotype-like sequences shown in panels B-D are outlined in black and labelled with the position of the SNPs included in the haplotype-like sequence. The haplotype-like sequences shown in panel E are not shown due to a missing corresponding single SNP sequence."

We have updated Figure S17 to highlight the differentially active haplotypes shown in 3C and 3E. The haplotypes shown in 3B and 3D were not labelled as differentially active and are thus not shown here. The updated version of Figure S17 is reproduced below:

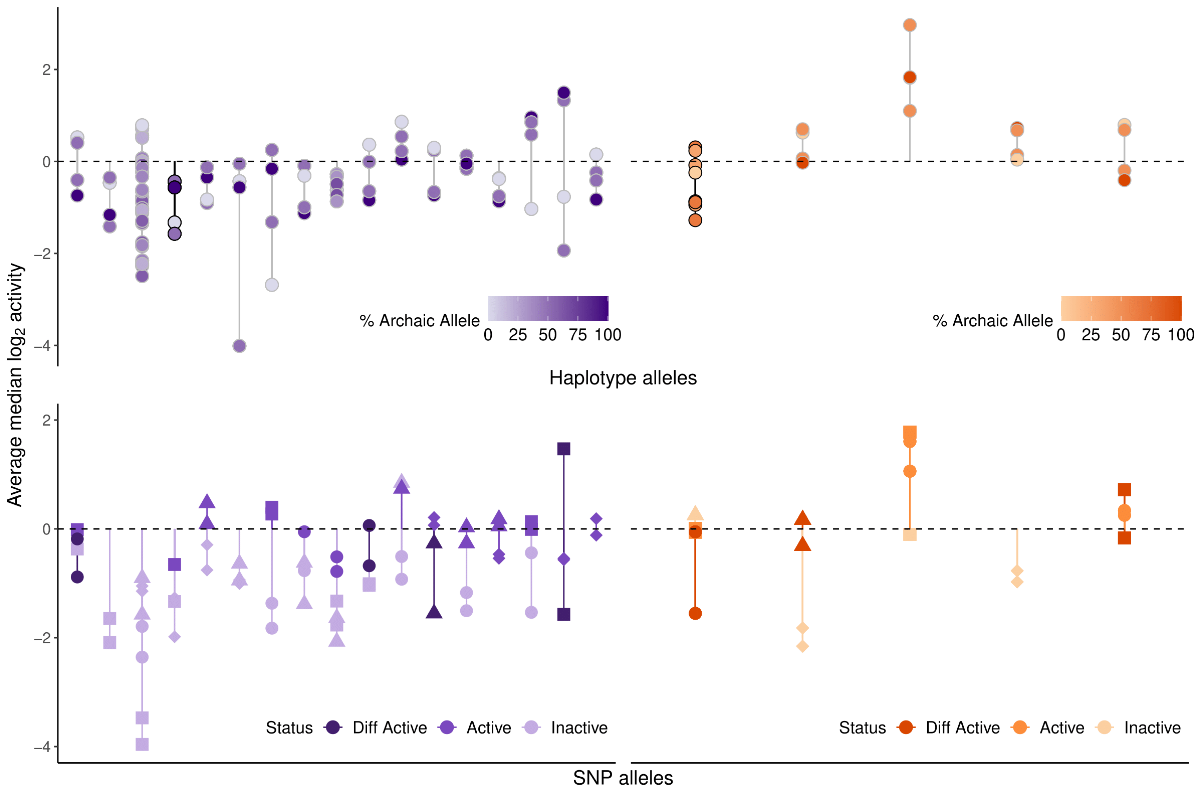

The caption for Figure S17 now reads:

“Activity of differentially active haplotypes (top row) and the activity of their corresponding single SNP sequences (bottom row) for Denisovan (left) and Neanderthal (right) sequences.} Haplotype-like sequences are coloured by the percentage of introgressed alleles within the haplotype. Pairs of shapes for single variant sequences represents the two alleles of any given SNP. The Denisovan haplotype shown in Figure 3C and the Neanderthal haplotype in Figure 3E are shown with black outlines.”
