# Supplementary material for: Mapping the gene regulatory landscape of archaic hominin introgression in modern Papuans
Source: PLoS Genet. 2026 Mar 24;22(3):e1012067. doi: 10.1371/journal.pgen.1012067 (PMC13012733; doi:10.1371/journal.pgen.1012067)
Supplement: Attachment — Submitted filename: Response_to_reviewers_auresp_3.pdf [file pgen.1012067.s038.pdf]

We thank the reviewer for their comments. We have elected to address the concerns raised in the discussion, including references to Abell et al., 2022 and Siraj et al., 2024 who have directly examined the effect of sequence context. Lines 489-500 now read:

"Finally, we observed moderate concordance between haplotype-like sequences and their constituent individual single-variant sequences under an assumed additive model. Our activity thresholding approach made interpreting the combinatorial effect of some variants challenging due to the fact that most MPRAs only detect activity above a baseline. Thus, when considering single-variants sequences that do not reach this cut-off, similar cDNA counts from two sequences can result in drastically different $\log_2$ activity estimates if pDNA levels are very different, even though they both indicate the same biological lack of regulatory potential. This makes additivity hard to interpret when considering inactive or lowly active sequences. Nonetheless, our results are broadly in line with those reported by other studies that have directly examined the effect of local sequence composition in expression levels using MPRAs, and found that the majority of effects between neighbouring variants are additive [72, 73]"
