# Supplementary material for: Mapping the gene regulatory landscape of archaic hominin introgression in modern Papuans
Source: PLoS Genet. 2026 Mar 24;22(3):e1012067. doi: 10.1371/journal.pgen.1012067 (PMC13012733; doi:10.1371/journal.pgen.1012067)
Supplement: Attachment — Submitted filename: Response to reviewers.docx [file pgen.1012067.s036.docx]

We thank the reviewers for their positive evaluation of our work. In response to comments from both reviewers we have made significant changes to the manuscript, including 10 new supplementary figures that support our overall conclusions, as well as added a number of statistical tests as requested. We have also reformatted the manuscript using the PLOS Genetics template, but provide a version with tracked changes using the older format. Line numbers below refer to the **newly formatted version**. Below, our responses and changes are indicated by blue text. New additions to the text are indicated by **bold blue text**.

Reviewer #1: The manuscript « Mapping the gene regulatory landscape of archaic hominin introgression in modern Papuans » by Comerford et al uses plasmid-based Massively parallel reporter assays to test the effect on lymphoblastoid expression of 25,869 introgressed alleles segregating at high frequency (>15%) in present-day Papuans. The manuscript is of high quality and combines general observations on the phenotypic effect of archaic introgression, with the identification of specific variants with strong effects on immune gene expression. The manuscript reads very well and is highly relevant to researchers interested in either genetics of gene expression, or population genetics.
I particularly enjoyed the results on the IFIH1 variant which I find to be a compelling example of how adaptive introgression has impacted the human immune system worldwide.

1/ Several analyses in the manuscript rely on comparing the percentages of active sequences across distinct classes of variants (eg. based on introgressed allele frequency or distance to TSS), however several comparisons are made without reporting significance, or confidence intervals.

For instance:
• Lines 177-178, the authors state that ‘More Denisovan active sequences were specific to the Papuan replicates (91 [out of 128] ) than Neanderthal active sequences (80 [out of 145])’. I’m not sure this difference is statistically significant. A Fisher’s exact test seems to give p=0.2.

Apologies for the confusion. There are 91 Denisova sequencies that are active only in the Papuan cell lines, and 128 that are active only in the GM12848 replicates. For the Neanderthal sequences, there are 80 that are active only in the Papuan cell lines replicates, and 145 that are active only in the GM12848 replicates. When testing for differences in the proportion of PNG-specific Denisova sequences and PNG-specific Neanderthal sequences, the difference is not significant (p = 0.378). However, when testing for differences in the proportion of GM12848-specific Denisova sequences and GM12848-specific Neanderthal sequences, the difference is significant, with a higher proportion of GM12848-specific active Neanderthal sequences. We have added this to the text, lines 137-143, which now reads as follows:

**When taking ancestry into account, we observed 145 Neanderthal sequences (out of 1,568 active Neanderthal sequences) and 128 Denisovan sequences (out of 2,059 active Denisovan sequences) that were active only in the GM12878 replicates, a significant excess of Neanderthal sequences (two sample proportion test p = 0.0007). Conversely, more Denisovan sequences were active only in the three Papuan replicates (91 out of 2,059) than Neanderthal sequences (80 out of 1,568) but the difference in this case was not significant (two sample proportion test p = 0.378).**

• Lines 196-197: “[the percentage of sequence located within 1kb of the nearest TSS that are active] is consistently lower for introgressed alleles than for AMH ones.” Could the authors provide a formal p-value?

And

More generally, several plots are hard to interpret due to the lack of confidence intervals, including figures 1E, 1F, 2C, 2D & S8. Confidence intervals could be easily computed with the binom.test R function.

While we do observe a lower percentage of active introgressed alleles within 1kb of the TSS than active AMH alleles within 1kb of the TSS, this difference was not significant. We have updated the text to reflect this, and have updated the surrounding text to better reflect our results after computing significance values and confidence intervals.

Lines 168 to 177 now read:

**“However, we observed a significant association between distance to the nearest annotated transcription start site (TSS) and the likelihood of a sequence being active (Figure 1F), with the percentage of active sequences being approximately 10% regardless of introgression source or allelic state when located > 1kb from the nearest TSS (Supplementary Figure 14B). Sequences located ≤ 1kb from a TSS were much more likely to be active ( χ 2 p = 2.2×10 −16 ), likely reflective of general genome architecture, as well of the limitations of the MPRA system to capture small effects typically associated with distant enhancers. When focusing on SNPs within 1kb of a TSS, we observe no effect of allelic state on the likelihood of activity for either Denisovan (logistic regression p = 0.149) or Neanderthal (logistic regression p = 0.673) sequences**”

We have also updated the discussion to reflect these results, lines 467-469 now read:

**“The percentage of active alleles did increase as distance to an annotated TSS decreased, but we did not observe an effect of allelic state (introgressed/modern) on the percentage of active alleles within 1kb of the TSS.”**

We agree that the plots can be strengthened with the addition of confidence intervals, but found it hard to incorporate these to the main figures as presented in the text. As such, we have tested whether the percentage of active/differentially active sequences in each bin/decile is different from the overall percentage of active/differentially active sequences (separately for Denisovan and Neanderthal sequences) and added the resulting confidence intervals to the current plots, broken down by introgression source, as Supplementary Figures 14 and 15. In general, we find that there are no differences between They are also reproduced here:

**Supplementary Figure 14**: Percentage active by A) allele frequency decile and B) distance to the TSS. For each decile/bin, we tested whether the percentage of active alleles is different from the overall percentage of active Denisovan or Neanderthal alleles (indicated by the dashed lines) with binom.test() and show the resulting confidence intervals.

**Supplementary Figure 15.** Percentage differentially active SNPs by A) allele frequency decile and B) distance to the TSS. For each decile/bin, we tested whether the percentage of differentially active SNPs is different from the overall percentage of differentially active Denisovan or Neanderthal SNPs (indicated by the dashed lines) with binom.test() and show the resulting confidence intervals.

• lines 213-216, the authors seem to oppose the pattern observed for Denisovan and Neanderthal alleles, yet they do not formally test for differences in the proportion of differentially active variants, with either one or both alleles between Denisova- and Neanderthal- introgressed SNPs.

We have updated these lines for clarity, as we were not aiming to contrast the patterns for Denisova and Neanderthal alleles. The text (lines 188-191) now reads

**“Of the differentially active Denisovan sequences, both the introgressed and non-introgressed allele were active in 54 cases, while the remaining 41 showed activity in one allele only. Of the 85 differentially active Neanderthal sequences, 35 showed activity in both alleles, with 50 showing activity in one allele only.”**

• Lines 234-236, the authors report higher percentage of differentially active SNPs among Denisovan-introgressed variants <1kb downstream of the promoter, but do not report significance.

We have updated lines 220-224 which now read:

**“However, we did observe an enrichment of differentially active Denisovan SNPs within 0.5 to 1kb downstream of the TSS compared to the overall percentage of differentially active Denisovan SNPs (binomial p = 0.008), suggesting that introgression with a functional effect can be tolerated within regulatory elements”**

2/ On a related note, the authors’ interpretation regarding the link between introgressed allele frequency and functionality is unclear, as several seemingly contradictory statements appear throughout the manuscript.

We thank the reviewer for raising these inconsistences. After calculation of confidence intervals and significance values, we have rephrased our results regarding allele frequency and activity to improve clarity and remove contradictory statements, as detailed in the next few responses.

• Lines 218-223, the authors claim that « Denisova and Neanderthal alleles display markedly different patterns », although as mentioned above, there is no statistical test to support a difference in the proportion of differentially active alleles between Denisovan and Neanderthal allele SNPs segregating at high/low frequency.

To support our statement, we have tested for an association between decile and percentage differentially active sequences separately for Denisova and Neanderthal sequences. We find that for Denisova sequences, the percentage of differentially active sequences is significantly correlated with decile, but do not identify any significant association for Neanderthal sequences. We have updated the text in lines 195 to 200 to reflect this, which now read:

**“The proportion of differentially active sequences is correlated with IAF decile for Denisovan sequences (Pearson's r = 0.710, p = 0.021), and increases from 3.7\% in the highest IAF decile to an average of 10.4\% for alleles in the bottom 60\% of the Denisovan sequences. IAF decile is less strongly associated with differential activity for introgressed Neanderthal sequences (Pearson's r = -0.027, p = 0.941).”**

• A few lines later (lines 229-230), the authors report a lack of significant correlations between introgressed allele frequency and logFC values (despite a suggestive trend for Denisovan-introgressed alleles at p=0.06 which they do not explicitly comment).

We have expanded our discussion of the association between IAF and logFC values to clarify our results. Lines 214 to 217 now read:

**“Despite the association between IAF decile and differential activity we find no statistically significant correlation between absolute log_2_ FC values of differentially active sequences and IAF in either ancestry (Spearman's ρ for Denisovan alleles = -0.192, p = 0.062; Spearman's ρ for Neanderthal alleles = 0.056, p = 0.61).”**

• In the discussion, (lines 442-445) they mention evidence of constraint against differential activity at high IAF for Denisova variants (despite the statistical significance of this observation has not been formally established, aside from the borderline signal at p=0.06) and discuss the lack of differentially active Neanderthal alleles at IAF>0.55, yet it is unclear whether the low number of Neanderthal alleles with IAF>0.55 could explain such a lack of active alleles.

While the number of alleles segregating at high IAFs (> 0.55) is lower than that of alleles with low IAFs, we believe this does not fully explain the lack of differential activity at high IAFs. We have updated the text (lines 208 - 212) to include the number of alleles at high IAFs tested for differential activity to clarify this. The text now reads:

**“Of the 56 Neanderthal SNPs segregating at an IAF >= 0.55 considered for differential activity testing, none are differentially active, while 1 Denisovan SNP with IAF >= 0.55 is differentially active (of 36 SNPs considered for differential activity testing) --- rs143481175, which is discussed further below and has the second strongest log2 FC in our data, -1.73, and an IAF of 0.64 in the Jacobs et al., 2019 dataset.”**

• In the abstract, the authors report no impact of introgressed frequency on activity, which seems in contradiction with the claims in lines 442-445.

We apologise for the confusion. In the abstract, we report on the association between IAF and activity, while in lines 442-442 we report on the association between IAF and differential activity. We have updated the abstract to make this distinction clearer, which now reads:

**“We found no association between introgressed allele frequency on activity regardless of introgression source, but introgressed Denisovan alleles at higher frequencies were less likely to be differentially active than expected, suggesting introgression is under some degree of selective constraint."**

I would suggest that the authors add an additional analysis where they test, for different frequency thresholds (eg. IAF>0.3, 0.4, 0.5, 0.55 & 0.6), how the percentage of differentially active sequences varies between alleles introgressed at high or low frequency (separately for Neanderthal and Denisovan alleles). Fisher’s exact test should be enough. If there is indeed a trend toward lower functionality at higher frequency for Neandertal/Denisovan-introgressed alleles, this analysis should grant them enough power to detect it.

We thank the reviewer for this suggestion, as it allowed us to confirm that there’s a depletion of differentially active Denisovan (but not Neanderthal) differentially active SNPs at higher allele frequencies. We have added this analysis as Supplementary Figure 16 (reproduced below), and updated the text in lines 203-208 which now read as follows.

**“In line with this, when testing for enrichment of differentially active sequences at varying IAF thresholds, we observe no enrichment or depletion for Neanderthal SNPs at any threshold, while we observe a significant deficit of Denisovan differentially active SNPs at IAFs >= 0.3 and 0.4 but not at >= 0.5 (although the point at 0.5 is also below 1, there are only 2 differentially active Denisovan SNPs with an IAF >= 0.5, explaining the large confidence intervals; Supplementary Figure 16).”**

**Supplementary Figure 16.** Enrichment of differentially active SNPs at varying introgressed allele frequency thresholds. Odds ratios calculated with Fisher’s exact test.

I would then encourage the authors to revise the text, in accordance with these new results to clarify their conclusions and avoid contradictory statements.

3/ I wonder to what extent the association that the authors find with the distance to TSS (Figure 1F) could be biased by the local GC content which tends to increase near the TSS? In my own experience, GC content can be linked with overall activity, with GC-rich regions often being more active. Have the authors looked into this? Do they see the same trend if activities are adjusted for GC content?

The reviewer is correct that sequence GC content can be linked to activity, as we do observe higher GC proportions in active sequences than in inactive sequences. We have added this in Supplementary Figure 10 (reproduced below) and in lines 148-150 which now reads:

**“Activity was associated with GC content, with active sequences showing higher GC content than inactive sequences (Denisovan Wilcoxon p = 3.21e-21, Neanderthal Wilcoxon p = 3.69e-22)”**

**Supplementary Figure 10.** Association between activity status and GC content for Denisova and Neanderthal sequences.

While we do observe a significant correlation between sequence GC content and absolute distance to the TSS, the magnitude of the effect is small (Spearman’s rho = -0.14, p < 2.2e-16). Thus, we believe the observed association with activity and distance to the TSS is robust to biases in GC content.

GC content can also be correlated with the number of barcodes associated with a specific sequence in the plasmid library, and thus the power to detect differentially active sequences. I don’t think this could explain the results from Fig 2D, but it could contribute to adding noise in the data.

The reviewer is correct that sequence GC content and number of barcodes is correlated (for archaic sequences, Spearman’s rho = 0.40, p < 2.2e-16). However, for a given SNP, the only difference in the two sequences is the allele of interest (i.e. the introgressed and non-introgressed allele are tested on the same background sequence). Thus, we do not think GC content is influencing the power to detect differential activity, as the only potential GC content difference comes from 1bp in the 200bp sequence.

Barcode GC content itself may contribute to the power to detect differential activity. To test this, we have calculated average barcode GC content for each oligo, and subsequently calculated the difference in average barcode GC content between the two alleles of each SNP. For Denisova and Neanderthal sequences, there is no difference in the distribution of barcode GC difference between differentially active and non-differentially active SNPs (Denisovan Wilcoxon p = 0.49, Neanderthal Wilcoxon p = 0.676). Thus, we do not believe that barcode GC is influencing our differential activity results.

4/ When discussing specific SNPs (eg. lines 325-327, or lines 379-389), the authors report a lack of associations for the reported SNPs in the GWAS catalog and instead report nearby GWAS associations as a proxy for gene function. While this approach is indeed justified by the general bias of GWAS toward Europeans, which prevents the interrogation of Papuan-specific variants, the authors could go a bit further by interrogating non-European Biobanks such as Biobank Japan (<https://pheweb.jp>) or the Taiwan Precision Medicine Initiative (<https://pheweb.ibms.sinica.edu.tw>), where some of these variants are present at reasonable frequency.

We thank the reviewer for their suggestions. We have added a discussion of the associations b between rs12464349 and phenotypes in BioBank Japan and TPMI in lines 345-351. The text now reads:

**“Although rs12464349 is not in the GWAS catalogue, likely due to its low global allele frequency, the surrounding region harbours multiple associations with immune phenotypes, including psoriasis (Figure 4C). rs12464349 is also associated with psoriasis phenotypes in the Taiwan Precision Medicine Initiative (TPMI) (Yang et al., 2024) catalogue and is associated with terms relating to kidney function in TMPI and Biobank Japan (Sakaue et al., 2021).”**

Meanwhile, rs143481175 is present in Biobank Japan at extremely low frequencies (ranging from 0.0009 to 0.034). There is a marginal association reported in that database (p = 1.5*10^-4) with “drugs for peptic ulcer and gastro-esophageal reflux disease” but given the low p-value and allele frequency we have elected not to report this in the text.

5/ While the analyses presented in Figure 3 are definitely interesting and appear rather sound, I found them difficult to understand. In particular, I regretted that the authors did not provide plots showing for specific loci the activity (y axis ) of each tested haplotype (x-axis), to highlight how the effect of the various SNPs combines in either an additive or non-additive manner.
Could the authors make plots (could be on the main figure, or in supplementary) where they focus on a single locus (i.e. one column of Figure 3D) to better illustrate the phenomenon they are discussing ?

To clarify my idea, I attached a toy example (Example_figure1.pptx) of the kind of plots I would expect. These could be shown in the supplement or in place of the current figure 3D (which could be moved to supplements to provide a fuller picture on haplotypic effects for the interested reader).

We have updated Figure 3 (reproduced below) and the text to illustrate examples of interactive effects between SNPs. We have moved Figure 3D to the supplementary materials, added new panels to Figure 3 (panels B-E) and added descriptions of the interactive effects we observed in lines 262-286, which now read:

**“We were able to identify haplotypes for which all alleles and corresponding single-variant sequences were active, but where the combination of alleles in the haplotype showed greater activity than that of the single-variant sequences alone (Figure 3B), suggesting additivity. We also identified single-variant alleles that alone appear to explain haplotype activity (Figure 3C-E). For example, the introgressed Denisovan allele at chr1 89,121,892 (rs149938333) is active in a single-variant sequence, and of the corresponding haplotype-like sequences spanning chr1 89,121,792 to 89,121,961, only those containing the Denisovan allele at rs149938333 are active. Finally, we observed instances where reconciling the observations at the individually allele level with the haplotype level was more challenging. For example, we tested a 3-SNP introgressed Neanderthal haplotype spanning chr1 38,030,248 to 38,030,417 (Figure 3E). When tested individually, at least one allele in all three SNPs was identified as active (the human allele at rs370339360 did not meet our quality control thresholds and data from it is not reported in the figure). However, at the haplotype level, only haplotypes containing the human allele at the second SNP, rs373127224, were active, regardless of allelic state at the two other SNPs. Our study design can explain this seeming contradiction: rs369802928 and rs373127224 are located 32bp from each other, while rs373127224 and rs370339360 are 52bp apart. As detailed in the methods, in our library single-variant oligos are centred on the variant of interest and flanked by 75bp of reference human genome sequence in either direction---which in this case always contains the human allele at rs373127224. Furthermore, rs373127224 is predicted to disrupt multiple transcription factor binding sites, while rs369802928 and rs370339360 do not overlap any. Thus, our data strongly suggest that out of the three SNPs tested, rs373127224 is the only one with the potential to drive activity, and highlight the value of combinatorial approaches such as these to fine map results.”**

As we moved Figure 3D to the supplementary materials, line 295-296 now reads: **“In this way we identified 22 haplotype sequences that showed differential activity at an FDR cutoff of 0.05 (Supplementary Figure 17)”**

The updated figure 3 is shown below.

**Figure 3: Testing of haplotype-like sequences.** A.) Schematic of haplotype-like oligo design. B-E.) Interactive effects between single variant sequences in haplotype-like sequences. F.) Activity of active haplotype-like sequences for which all corresponding single SNP sequences are inactive for Denisovan (top) and Neanderthal (bottom) sequences. Plots are ordered by decreasing difference between haplotype and summed SNP activity. G.) Correlation between activity of haplotype-like sequences and the summed activity of the corresponding single SNP sequences for Denisovan (left) and Neanderthal (right) sequences. Mixed activity of summed single SNP sequences represents cases where the single SNP sequences for a given haplotype sequence were different in their activity status.

6/ It seems that the assignment of target genes is mainly performed based on distance and known gene annotations (for TNFAIP3). Did the authors consider validating the targets of the identified variants through CRISPR or equivalent? Alternatively, they could use Promoter-capture HiC data from (Javierre et al., Cell, 2016), or HiChIP data from (Chandra et al., Nat Genet., 2021). They could also query the SCREEN database (<https://screen.wenglab.org/>) to identify putative enhancer-gene links based on experimental evidence (Intact Hi-C Loops, ChIA-PET Interactions, CRISPRi-FlowFISH), or computational predictions (ABC, EPIraction, GraphRegLR, or rE2G).

We thank the reviewer for their suggestions. While we did consider CRISPR and manually examined alleles on an ad-hoc basis against public Hi-C maps, we ultimately decided that systematic application of these approaches was outside our capabilities. Linking of genes and enhancers at scale remains challenging and no method is perfect—this of course includes nearest gene approaches, although they are not significantly less accurate than other approaches (see, e.g., Wang and Goldstein, AJHG, 2020). However, on the SCREEN database rs143481175 is predicted to lie within a proximal enhancer that is linked to *TNFAIP3* and nearby lncRNAs, but not *OLIG3*, in CD4+ T-cells. This is now reported in the text, on line 377-379:

**“In the SCREEN database, rs143481175 is predicted to lie within a proximal enhancer that is linked to *TNFAIP3* and surrounding lncRNAs, but not *OLIG3*, in CD4+ T-cells.”**

7/ Although I would not make this a strict requirement, I think the manuscript would be strengthened if the authors could validate some of their main findings (eg. rs12464349, & rs143481175) through standard luciferase assays to demonstrate that their main findings are robust and replicate with a different technique.

We agree with the reviewer that replication of differentially active SNPs would be useful in validating the MPRA approach, and considered how we might provide orthogonal validation of our results. Fundamentally, an MPRA is just a lot of luciferase assays together with a sequencing barcode, and so we are not convinced this would provide additional information over our five experimental replicates and many barcodes. Furthermore, we’d be limited to one cell type—LCLs—and it’s not clear that this is the most relevant cell type!

Instead, to support our findings across multiple cell types, we generated computational predictions of variant effect using the state of the art sequence-to-function model AlphaGenome (Avsec et al, bioRxiv, 2025). Because these kinds of models struggle to predict long-range impact on gene expression, we predicted the effect of our top differentially active SNPs (rs12464349 and rs143481175) on chromatin accessibility (ATAC-seq) across a range of immune cell types. We have added these results to Figure 4 (Figure 4I), as well as summarised predictions across all differentially active alleles to the supplementary materials (Supplementary Figure 23) and have updated lines 420 and 434, reproduced below. Overall, while we agree with the reviewer that experimental replication of results is valuable , we believe that our computational analysis support and extend our MPRA findings.

**“Finally, we generated computational predictions of variant effect using AlphaGenome (Avsec et al., 2025). Because AlphaGenome struggles to predict effects on gene expression levels as distance to the target gene increases, we instead predicted ATAC-seq read coverage, as a proxy for chromatin accessibility, for all SNPs tested for differential activity across a range of immune cell types. Predicted ATAC-seq score was correlated with mpralm log_2_ FC across immune cell types for both Denisovan and Neanderthal SNPs (Denisovan Spearman's ρ = 0.112 to 0.2, p $<$ 0.001; Neanderthal Spearman's ρ = 0.114 to 0.204, p $<$ 0.001; Supplementary Figure 23). When focusing on the regions surrounding our top two differentially active SNPs, both rs12464349 and rs143481175 had predicted ATAC-seq scores of greater magnitude than SNPs in the surrounding ± 1Mb regions (rs12464349 t = -3.7365, p = 0.002; rs143481175 t = -6.263, p = 6.95 x 10^-5^; Figure 4I). The direction of allelic effect was consistent with our MPRA results, with the introgressed allele for both SNPs predicted to have greater ATAC-seq coverage than the modern human allele, providing orthogonal evidence for a role for these sites in gene regulation.”**

Both figures are reproduced below.

**Figure 4**. Differentially active SNPs are predicted to regulate immune genes. A.) Activity of rs12464349 alleles across replicates. B.) Allele activity difference for SNPs surrounding rs12464349. The transcription start site of IFIH1 is indicated with a dashed line. C.) GWAS catalogue hits surrounding rs12464349 (indicated with a red line). D.) Introgressed allele frequency for rs143481175:C in the HGDP-CEPH populations, taken from gnomAD v 4.1. The pie centred over Port Moresby, Papua New Guinea, illustrates allele frequency in genetically Papuan individuals from Vespasiani et al., 2022. E.) Allelic states in archaic humans and the African Yoruba (YRI) population, together with the haplotype structure across archaic marker SNPs associated with archaic haplotypes surrounding rs143481175 (bolded position) in samples from Yermakovich et al., 2024. F.) Allele activity difference for SNPs surrounding rs143481175. The transcription start site of TNFAIP3 is indicated with a dashed line. The positions of rs376205580 and rs141807543 are indicated with black lines. G.) Activity of rs143481175 alleles across replicates. H.) Distribution of barcode level activity for haplotype-like oligos containing rs143481175 and corresponding single-variant oligos across replicates. Numbers indicate the number of barcodes associated with each sequence. I.) AlphaGenome ATACseq predictions for rs12464349 (left; bolded position) and rs143481175 (right; bolded position) and the surrounding SNPs tested for differential activity across immune cell types. AlphaGenome ATACseq score refers to the effect of the introgressed allele relative to the non-introgressed allele.

**Supplementary Figure 23**. Association between mpralm logFC and AlphaGenome ATACseq score across immune cell types.

*** Minor comments: ***

1/ line 54: the authors report that Denisova introgression accounts for up to 5% of genomes of Papuans and Near Oceanic Ancestry. I believe this estimate is outdated and more recent studies (eg. Choin et al, Nature, 2021) have estimated that while Papuans indeed carry ~5% of archaic DNA, only 3% of their DNA is inherited from Denisovan, with the remaining 2% being inherited from Neanderthals through admixture during the Out-of-Africa. I would suggest replacing « up to 5% » with « an additional 3% ».

We have made the suggested replacement, and additionally cited Choin et al., 2021. Lines 9-11 now read:

**“while Denisovan introgression accounts for an additional 3\% of the genomes of individuals of Papuan or Near Oceanic genetic ancestry (Reich et al., 2010; Meyer et al., 2012; Choin et al., 2021).”**

2/ In Figure 1B, the authors show the number of Vindija/Denisova introgressed alleles falling in open/active chromatin regions and /or disrupt a transcription factor binding site. To my surprise, however, I did not see a breakdown of the percentage of active/differentially active CRS according to the SNP annotation (open/active chromatin & TF disruption. This should be easy to assess and would be very useful for the design of future MPRA experiments. Could the authors provide plots similar to Fig S8A and S8B, but grouping SNPs with the various combinations from Fig 1B?

We agree with the reviewer that these annotations will be useful for guiding variants to include in future MPRA experiments. We have added these figures as Supplementary Figures 11 and 12, and updated the text in lines 151-154 and 226-227 which now read:

**“In addition, active sequences occurred in all regulatory annotation classes (Supplementary Figure 11A and B). There was no significant association between immune cell specific annotations and activity, but active alleles were enriched in open chromatin annotations shared across cell types and depleted from closed chromatin (Supplementary Figure 12).”**

and

**“Indeed, we observe the highest proportion of differentially active SNPs for SNPs predicted to disrupt TF binding (Supplementary Figure 11C and D)”**

**Supplementary Figure 11**. Percentage A-B) active sequences and C-D) differentially active SNPs across the annotations used to define SNPs for testing (from Figure 1B). Active and open refer to active chromatin and open chromatin, respectively.

3/ In figure S1 & S2, the total number of SNPs does not seem to match across panels (eg. between B cells and LCLs). Is there an explanation for this? Or is this an error?

No this is not an error. A SNP might be annotated with multiple states in B cells, for example, while only being annotated with one state in T cells, which will inflate the B cell counts. This means the total number of SNPs will not necessarily match across panels.

We have clarified this in the text, lines 551-554, which now read: **“SNPs annotated in at least one active chromatin state within monocytes, LCLs, B or T cells in Epigenome Roadmap data (Kundaje et al., 2015) were retained for testing (Supplementary Figures 1 and 2). SNPs may be assigned to multiple annotations within and/or across cell types.”**

4/ In figure S4, could the authors clarify how the number of barcodes per oligo is computed. Is it the total number of barcodes associated with a specific oligo at the association-sequencing step? Or the number of barcodes present in the plasmid-pool and with non-zero RNA reads in at least one replicate? if the same barcode is present in multiple replicates, is it counted twice, or only unique barcodes are counted?

We have clarified how the number of barcodes per oligo is calculated in the caption of Supplementary Figure 5 (previously S4), which now reads: **“Distribution of barcodes per oligo post filtering. The number of barcodes is calculated as the number of unique barcodes with average pDNA log2CPM > -5 across replicates.”**

5/ In figure S7, the correlation between replicates appears rather low, which I suspect is due to sequences with a low number of associated barcodes driving the correlation downwards. Could the authors repeat the analysis from figure S7A, stratifying by number of barcodes.

We agree with the reviewer that the correlations presented in figure S7 are driven by sequences with a low number of associated barcodes. We have repeated the analysis presented in Figure S7A stratified by the number of barcodes and added this as a new supplementary figure (Supplementary Figure 9).

This is now mentioned in the text in line 131.

We note that we do not expect this poor correlation at low barcode levels to give rise to false positive results, due to how we define activity and differential activity. It does suggest, however, that we might be somewhat underpowered to detect small changes across alleles.

In addition, see our reply to reviewer 2, point 4, where we have also examined the correlation at the oligo level (summed across all barcodes).

**Supplementary Figure 9**. Spearman’s correlation of median activity across replicates for all sequences after filtering for sequences with at least A) 10, B) 20, C) 30, D) 40, E) 50, F) 75 and G) 100 barcodes. The number of sequences retained after each barcoding threshold is displayed.

Reviewer #2: Here, Comerford and co-authors present results from using a massively parallel reporter assay to evaluate regulatory activity across a panel of nearly 26k variants with alleles from different introgression sources (Neanderthal, Denisovan) and states (introgressed, modern) in individuals of Papuan ancestry. This library is tested in several lymphoblastoid lines of different ancestries, and the authors find that in each of the Denisovan and Neanderthal sequence sets, around 8-9% display enhancer activity. Within these active sequences, ~9% have differential activity across modern and archaic alleles.

Overall, I’m enthusiastic about the motivation of the study and results presented here; however, there are several points that could be addressed to enhance the study’s impact. I will note that I have expertise in functional genomics/MAVEs and not in evolutionary biology, so my comments will mostly focus on the former.

Major:
1. I appreciate the use of multiple LCLs representing multiple ancestries. I was wondering why you used two replicates per cell line versus >2 replicates in one or both of the Papuan lines. I understand the value of looking across ancestries, but then that feature of the experimental design is never really commented on or addressed in the results section.

We agree with the reviewer that examining the impact of the trans environment on gene expression is indeed valuable, and that was our motivation for including multiple cell lines with different genetic backgrounds. However, given our number of replicates and the relatively small effect size of most trans changes identified to date by other researchers we are not confident that we have enough power to identify robust differences beyond what’s detailed in lines 137-143, reproduced below, and thus chose not to pursue this further until we have a better-powered study design.

**When taking ancestry into account, we observed 145 Neanderthal sequences (out of 1,568 active Neanderthal sequences) and 128 Denisovan sequences (out of 2,059 active Denisovan sequences) that were active only in the GM12878 replicates, a significant excess of Neanderthal sequences (two sample proportion test p = 0.0007). Conversely, more Denisovan sequences were active only in the three Papuan replicates (91 out of 2,059) than Neanderthal sequences (80 out of 1,568) but the difference in this case was not significant (two sample proportion test p = 0.378).**

This observation does suggest that there is possibly more to uncover here, and it is something we hope to come back to in future work!

2. You mention the relatively low pairwise correlation of activity you see between replicates which improves when you filter to your positive control set. Have you also looked at pairwise correlation between pDNA replicates? My expectation is that it should be pretty high given that you’re delivering the same library to all samples.

The reviewer is correct. Correlation between pDNA replicates is high (~0.9 for each pairwise comparison). We have updated the text (lines 103-105) with reference to these values. The text now reads as follows:

**“Correlations between the pDNA counts across replicates (Spearman’s rho = 0.90 - 0.91), were higher than that of the correlations between the cDNA counts (Spearman’s rho = 0.59 - 0.70). Sequences counts were moderately correlated between the cDNA and pDNA fractions within replicates...”**

3. You include a set of 300 negative control sequences in your library, but then use the mean cDNA/pDNA counts within a replicate as your threshold for calling sequences active/inactive. Did you consider using your negative controls to estimate a null distribution for this testing instead?

While we did consider using the negative controls to estimate a null distribution, within each replicate the mean of the negative control sequences is similar to the mean of all sequences used as our threshold for activity testing (see Table 1). As such, we do not expect our activity calling results to change substantially if using the negative control sequences to establish a null distribution for testing.

| Group | GM12878 Rep1 | GM12878 Rep2 | PNG22 Rep1 | PNG22 Rep2 | PNG8 Rep2 |
| --- | --- | --- | --- | --- | --- |
| Denisova | -0.6689094 | -1.6797918 | -3.503793 | -2.0061487 | -2.522238 |
| Neanderthal | -0.6533932 | -1.6593622 | -3.477224 | -1.9761401 | -2.496291 |
| Tewhey | 0.3074991 | -0.1210595 | -2.038775 | -0.5195537 | -0.977236 |
| Arensbergen | -0.1155417 | -0.2983454 | -2.642980 | -1.0677620 | -1.630738 |
| Random (negative control) | -0.6838725 | -1.6452638 | -3.588318 | -2.0879967 | -2.616717 |
| Overall | -0.6501280 | -1.6447242 | -3.473577 | -1.9723380 | -2.490830 |

Table 1: Mean activity across replicates for each sequence type in our library.

4. Could you clarify your rationale for using barcode-level estimates of activity for statistical testing within each replicate? Because your replicate correlations were somewhat low, I’m not sure that this approach adequately captures this variability.

When we first began this project we initially followed the two-step approach of Uebbing et al, PNAS, 2021. These authors first use a one-tailed t-test to establish activity above the baseline for a given oligo across barcode but within replicates, as we have done, and then a second two-tailed t-test that compares activity across all barcodes of each allele for a given a oligo. The purpose of the first t-test is to moderate the number of tests for differential activity, because there is no point in testing alleles where there is no power to detect differential activity.

In our mind, the first t-test is analogous to removing lowly expressed genes from RNA-seq datasets before testing for differential expression, which is why we applied it. However, rather than use a two-tailed t-test for establishing differential activity, we used mpralm, which takes advantage of the limma/voom framework for explicitly modelling count data. Importantly, data for differential expression testing with mpralm is not modelled at the barcode level, but rather summed across all barcodes associated with an oligo, following the recommendations in the Myint et al paper. Thus, our differential activity testing should be robust to variation at the barcode level.

It’s worth noting that we do see significantly higher replicate correlations at this level, as could be expected and is now shown in a new panel in Supplementary Figure 8:

This is now directly discussed in the text, lines 128-131:

**Activity ratios were moderately correlated across replicates and, as expected, were substantially higher when considering summed activity (Supplementary Figure 8A). Activity of the positive control sequences showed the highest correlation of all sequence types (Supplementary Figure 8B-E, Supplementary Figure 9).**

At the time of transfection our library contains an average of 122 barcodes per oligo. Low correlations in activity between replicates is driven by sequences with relatively fewer number of barcodes (addressed in Reviewer #1 minor comment 5 and new Supplementary Figure 9).

5. As a related question: with the exception of your positive control sets, the two GM LCL replicates have a consistently higher proportion of uniquely active oligos compared to the other two lines (Supplementary Figure 6). I assume this is just a thresholding effect where marginal cases aren’t captured, but I wasn’t sure if there was some other interpretation

We also noticed this, yes. It’s hard to say what is causing it—it might be that transfection of the two GM12878 replicates was more efficient in general and thus this is attributable to technical variation between lines—this is not completely unheard of in the case of MPRAs. All replicates contained similar numbers of cells and were processed at the same time; they were also sequenced to similar depths, so we have no better answer to offer!

6. More clarity on how you performed the differential activity testing would be helpful. To identify active oligo sets, you’re performing statistical testing within each replicate and then defining a consensus set across replicates (FDR < 0.05 in at least two). Are you using data across all 5 replicates to perform differential activity testing? You include the model you pass to mpralm, but it would also be helpful to know what the design vector (i.e., other covariates) looks like.

The reviewer is correct, yes. We ask that the one-tailed t-test be significant in at least 2 replicates to define the set of active oligos, but data from all five replicates is passed on to mpralm when testing for differential activity. Oligos with highly variable behaviour across replicates are unlikely to reach statistical significance in our differential activity testing, since the within-group variance will be high and decrease our power to call differential activity.

The only other covariate in the model is the genetic background of the cell line. We have clarified this in the relevant methods section, which now reads (line 667-676):

**“All sequences with at least one active allele were then tested for differential activity. Where relevant, we compare active alleles to their inactive counterparts. We take two approaches to differential activity testing. First, to test for differential activity between two alleles of a SNP, we used the mpra v1.26 R package (Myint et al., 2019), which makes use of the voom framework (Law et al., 2014) to model the properties of count data. As recommended by (Myint et al., 2019) cDNA and pDNA counts for each oligo were summed across barcodes and values for all five replicates passed on to mpralm. The model included terms for allelic state (introgressed or not) and cell line genetic background (Papuan or not). We define sequences as differentially active between the introgressed and modern alleles if it has a Benjamini-Hochberg adjusted p-value < 0.05.”**

7. You used regulatory annotations to help select the variants tested in the library. You mention that the regulatory annotations are defined in at least one immune cell type – I assume this means that some of your annotations are unique to one or more immune cell types while others are shared across lots of cell types. Are there any worthwhile insights to be gained if you partition the regulatory annotations based on whether they’re unique to immune cells versus shared broadly across a lot of cell types?

Yes, as a result of our selection criteria, some SNPs have regulatory annotations unique to our immune cell types of interest (B cells, LCLs, monocytes and T cells). However, generally, the annotations are not unique to immune cells and are shared with other cell types. We have added Supplementary Figure 3showing the specificity of annotations to immune cell types, and updated lines 73-76 as follows:

**“We did not require the annotations to be unique to immune cells; indeed, most annotations were shared across at least one immune and one non-immune cell type (Supplementary Figure 3)”**

To test whether specificity of regulatory annotations influence’s activity, for each allele we define whether it is annotated in active chromatin specifically in immune cells, in non-immune cells, both or neither. Likewise, we define whether an allele is annotated in an ATAC or DHS peak specifically in immune cells, specifically in non-immune cells, both or neither. For these annotations, we test for an association with active status and differential activity status. We find no association between chromatin state annotation and active status for Denisovan or Neanderthal alleles. We do observe an association between peak annotation and active status for both Denisovan and Neanderthal alleles. We have added these results as Supplementary Figure 12 and have updated lines 152-154 to discuss these results. These lines now read as follows: **“There was no significant association between immune cell specific annotations and activity, but active alleles were enriched in open chromatin annotations shared across cell types and depleted from closed chromatin (Supplementary Figure 12).”**

**Supplementary Figure 3.** Cell type specificity of annotations used for SNP selection for A) Denisova and B) Neanderthal SNPs**.**

**Supplementary Figure 12**. Enrichment of active sequences across SNP annotations, stratified by whether that annotation cell type specific or shared.

8. Your transcription factor binding site results are really exciting! Are you restricting your analysis to only TFs that are expressed in LCLs/immune cells broadly, or any TFs?

We share the reviewer’s excitement with regards to the TFBS analysis. We did not apply any restrictions to the transcription factors included in the analysis, and have clarified this in lines 704-706 which now reads:

**“The trimmed list of sequences was passed to FIMO (Grant et al., 2011) to identify matches with the HOCOMOCO v11 core collection (Kulakovskiy et al., 2018), regardless of their expression in LCLs or immune cells, and only retaining matches with p-value < 0.001 in the output.”**

9. In your haplotypic analysis, you see that simply summing the effects of individual alleles doesn’t fully capture the activity of the full haplotypes. I am very excited by this subset of the library and think this is sorely lacking from a lot of MPRA studies. It would enhance the impact of this section if you could elaborate on these results a bit more – e.g., do you find any examples where there are clear driver variant(s) that seem to drive the synergistic effects? Is there anything interesting/unique about variants that fall off the diagonal in Figure 3C (top left, bottom right quadrants)?

In response to this comment and a similar one from Reviewer 1 we have restructured this section to examine specific examples in a bit more depth, and changed the panels in Figure 3, reproduced below. There is also additional discussion of some of the observed trends in lines 262-286.

**Figure 3: Testing of haplotype-like sequences.** A.) Schematic of haplotype-like oligo design. B-E.) Interactive effects between single variant sequences in haplotype-like sequences. F.) Activity of active haplotype-like sequences for which all corresponding single SNP sequences are inactive for Denisovan (top) and Neanderthal (bottom) sequences. Plots are ordered by decreasing difference between haplotype and summed SNP activity. G.) Correlation between activity of haplotype-like sequences and the summed activity of the corresponding single SNP sequences for Denisovan (left) and Neanderthal (right) sequences. Mixed activity of summed single SNP sequences represents cases where the single SNP sequences for a given haplotype sequence were different in their activity status.

With regards to the points off the diagonal, this is a good question, and one we had not given much consideration to. We have now examined the oligos/regions in the two quadrants off the diagonal, and find that some are consistent, but our scale makes them look not so. For instance, in this 2-SNP haplotype, the first SNP is active regardless of allelic state, and the second SNP is inactive regardless of allelic state. All four haplotype SNPs are active, suggesting that the first SNP is driving the signal here. However, because the activity of the first SNP on its own is ~0 and that of the second SNP is ~ -2, the sum of activities (x axis in Figure 3C) is –2, whereas the haplotype activity is slightly greater than 0, and not that different from the activity measurement of the oligos containing only the first SNP. Indeed, negative activity levels are noisy and have a high degree of stochasticity, especially given that our assay design can only detect activation **above** a baseline; additivity is harder to interpret when combining negative and positive values. In this case, this region is not that different from the one we display in Figure 3E, except for the actual axis values.

There is also a second class of regions, shown below:

In this case, all alleles tested individually are inactive, but all haplotypes are clearly active, and there’s a big difference in median activity between the two kinds of oligos. The biggest difference between the two classes of oligos is that haplotype-like oligos are centered on the haplotype midpoint, whereas single SNP oligos are centered on the SNP. This means that the sequence being tested between the two is subtly different. We hypothesise that in some cases this might lead to the gain or loss of transcription factor binding sites or other functional elements, which account for the observed patterns. However, because this is fairly speculative we have elected not to add it to the text at this point.

Minor:
1. Can you add titles to Figure 1B to make it very explicit what purple and orange are since it’s the first time you use this color scheme? It’s nicely labeled elsewhere.

Titles have been added to Figure 1B.

2. Line 258: what percentage of all haplotype-like sequences that were designed does this represent?

We have updated line 249 with this value. The text now reads:

**“Although all allele combinations were included in the originally synthesised oligonucleotide pool, only 6,537 haplotype-like alleles (72.99\% of haplotype-like alleles in the initial library) were considered for activity and differential activity testing.”**

3. I appreciate the insights presented in Figure 4H, but I find this figure a little bit hard to follow. I generally read figures from top-to-bottom, so I think it could be helpful to flip the order of results presented on the y-axis which also aligns with how you talk about these data in the results section: first single-SNP oligos, then haplotype-like oligos.

We have changed Figure 4H to reflect the order of the results in the text.

4. The observations presented in lines 469-473 are striking/disappointing, and really point to the importance of the work you’re presenting here. I completely agree with your claims in the final lines of the discussion (“MPRAs and other modalities of multiplex assays of variant effects (MAVEs)...” I’d recommend reiterating your findings in lines 294-297 to provide further validity – it’s not just about the feasibility of MAVEs versus generating large-scale datasets in non-Europeans, but also that they’re quite accurate!

We thank the reviewer for this suggestion, and have updated the discussion to reiterate our GTEx eQTL overlap results. The section now reads (lines 513-527):

**“For example, when looking for overlap between variants in the GWAS catalogue (Sollis et al., 2023) and our active SNPs, only 2 Denisovan and 5 Neanderthal SNPs were present in the GWAS catalogue. We were able to identify 274 active SNPs that are also eQTLs in GTEx v8 (The GTEx Consortium, 2020), the majority of which were Neanderthal hits, as expected due to the disproportionate number of European donors in GTEx. These disparities have been well-documented before (e.g. Sirugo et al., 2019; Gallego Romero, Rodenberg, et al., 2024); both logistic and ethical challenges in generating these datasets outside the Global North (George et al., 2023) mean that they will be hard to redress in the short and middle term, at least at comparable scales to existing resources. Within this set of overlapping SNPs, however, the direction of effect was generally consistent between GTEx and our MPRA, confirming the value of MPRAs for large-scale variant effect testing and fine-mapping. Thus, MPRAs and other modalities of multiplex assays of variant effect (MAVEs), alongside computational predictors of variant effects, emerge as leading candidates to drive our understanding of the functional consequences not only of introgressed variants, but of genetic variation found in groups and individuals who are poorly represented in existing resources.”**
